# Identification and dietary exposure assessment of tetracycline and penicillin residues in fluid milk, yogurt, and labneh: A cross-sectional study in Lebanon

**DOI:** 10.14202/vetworld.2019.527-534

**Published:** 2019-04-14

**Authors:** Suzanne Kabrite, Christelle Bou-Mitri, Jessy El Hayek Fares, Hussein F. Hassan, Jocelyne Matar Boumosleh

**Affiliations:** 1 Department of Nursing and Health Sciences, Notre Dame University, Louaize, Zouk Mosbeh, Lebanon; 2 Department of Natural Sciences, School of Arts and Sciences, Lebanese American University, Beirut, Lebanon

**Keywords:** antibiotic residues, dairy products, enzyme-linked immunosorbent assay, estimated dietary intake, penicillin, tetracycline

## Abstract

**Background and Aim:**

The safety and quality of dairy products are considered to be of significant importance to human health. Although antimicrobial drugs are essential for disease treatment in modern medicine, the use of these drugs can have undesired consequences for human and animal health. This study aimed to investigate the presence of tetracycline and penicillin residues in raw, pasteurized, and UHT cow’s milk of different fat contents, as well as in the dairy products yogurt and labneh, a traditional Lebanese product.

**Materials and Methods:**

A total of 44 samples, 4 raw, 9 UHT, 9 pasteurized milk, 10 yogurt, and 12 labneh samples from common local brands available in the Lebanese market were collected from Keserwan regions in May 2016. Tetracycline and penicillin residues were determined using a competitive enzyme-linked immunosorbent assay (ELISA) technique.

**Results:**

The mean values for tetracycline and penicillin were all below the limit of detection (LOD) of the ELISA kit of a maximum standard concentration of 1.80 µg/kg and 4.00 µg/kg, respectively. All samples tested positive for antibiotic residues. The detection rate for tetracycline in milk (n=22) samples was 86.4% with a mean residues value of 1.16±0.70 μg/kg. The detection rate of tetracycline in labneh (n=12) and yogurt (n=10) samples was 50% for each with a mean value of 1.76±0.40 μg/kg and 0.63±0.12 μg/kg, respectively. As for penicillin residues, 90.9% of the milk (n=22) samples tested positive with a mean value of 0.52±0.25 μg/kg. The detection rate in labneh (n=12) and yogurt (n=10) samples was 0% for penicillin residues, where mean values were all below the LOD (<1.25 μg/kg) for these dairy products. None of the samples exceeded the maximum residue levels. The estimated dietary intake (EDI) for tetracycline and penicillin residues for all dairy products is 2.09 ng/kg body weight (BW)/day resulting in 0.007% of the acceptable daily intake (ADI) and 1.83 ng/kg BW/day resulting in 0.006% of the ADI, respectively.

**Conclusion:**

All EDI values were below the ADI set for each antibiotic residue and do not exceed relevant toxicological reference values. However, concerns might still be present from consumption of other animal food products containing residues. Moreover, the long-term exposure to such residues is still unknown as a result of bioaccumulation; it is a challenging process to determine the actual dietary consumption of foods containing antibiotic residues; hence, the human health risk cannot be easily predicted.

## Introduction

The safety and quality of dairy products are considered to be of significant importance to human health [1]. Consumers have the right to be confident that their food supply, including dairy products, is free of contamination. Foodborne illnesses account for substantial morbidity and mortality worldwide [2]. The most common foodborne illnesses, caused by viruses, parasites, and bacteria, are among the major causes of diarrhea and related complications such as kidney failure, abdominal pain, and dysentery as well as death [2]. Antimicrobial drugs have played a major role in minimizing illnesses and deaths associated with infectious diseases in animals and humans [3,4]. In addition to their traditional use as a treatment for bacterial infections, antibiotics are used in agriculture both as growth-promoting substances and as prophylactic additives to prevent the occurrence of diseases [5]. Worldwide, about 50% of all antibiotics made are used in animal agriculture applications [6]. Tetracyclines and β-lactam group are among the most common veterinary classes of drugs used in dairy cattle breeding systems worldwide [1,7]. Tetracyclines are broad spectrum and the most commonly used antibiotics for prophylactic and growth promotion purposes in many countries around the world, with oxytetracycline (OTC) being one example [8]. In addition to tetracyclines, β-lactam group of antibiotics is widely used in therapy for bacterial infections in cattle, particularly for the treatment of mastitis, the most common disease in adult dairy cows [9,10]. Among antibiotics in this group is penicillin, which is one of the oldest and most widely used antibiotics in the treatment of Gram-positive and Gram-negative bacterial infection in humans and animals [11]. The use of antibiotics has been regulated since the 1990s, and maximum residue limits (MRLs) have been established for a variety of antibiotics for different animal tissues, milk, and eggs [12]. According to the European (EU) and Codex Alimentarius Commission standards (CAC), the MRL in milk for tetracyclines, including OTC, is 100 µg/kg (100 ppb), whereas the MRL for β-lactams is specific to the antibiotic; for example, the MRL for penicillin is 4 µg/kg (4 ppb) [7,13]. Milk is continuously controlled for the presence of antimicrobial residues according to the legislative requirements in many countries [10]. However, antibiotic residues are still reported to be present in milk in levels exceeding the MRLs in different regions around the world due to poor management practices, economic factors, and farmers’ lack of awareness and education [1,14-16]. The risk of consuming milk containing antimicrobial residues, even when present below the MRL, is also of great concern to human health. The long-term bioaccumulation of antibiotic residues may result in bacterial resistance [5,9], hypersensitivity reactions [9,17], gastrointestinal and liver problems [15], as well as triggering cancer, mutagenicity, and toxicity in humans [18].

In Lebanon, dairy products are considered an essential part of the Mediterranean diet with the average intake of dairy products among adults living in different areas reported to be ranging between 243.1 g/day [19] and 350.5 g/day [20], which is considered to be very close to other neighboring Mediterranean countries [21] and comply with the guidelines for the Mediterranean region for dairy products of 1-2 servings/day (where 1 serving is equivalent to 1 cup or approximately 230 g) for the intake of Lebanese population. Moreover, based on country-specific estimates of per capita milk consumption, Lebanon is classified to be among the countries which have a high intake of milk defined as per capita milk consumption/year of >150 kg [22]. Lebanese dietary intake includes a variety of dairy products including fluid milk, yogurt, and, the traditional dairy product, labneh (strained yogurt) [19], and since the Mediterranean diet emphasizes on choosing low fat and skimmed dairy products, it is necessary to take into consideration different fat content of each product when assessing the levels on antibiotic residues. Moreover, a study conducted by Kassaify *et al*. [23] confirmed the presence of resistant strains of major pathogens against two antibiotics gentamicin and streptomycin in raw milk samples in Lebanon, and samples containing residues below the MRL for these antibiotics were reported. The lack of accurate data on the usage of antibiotics necessitates the investigation of the potential presence of other antibiotic residues in milk which could aggravate the growing threat of antibiotic resistance, especially that antimicrobial resistant pathogens that were found in bovine milk may be the result of the excessive and extended applications among farmers [23].

In light of the above, exposure assessment to antibiotic residues in dairy products has become a primordial step for risk assessment in the Lebanese population. Therefore, the objective of this study was to assess the level of exposure of the Lebanese population to tetracycline and penicillin found in dairy products and to determine accordingly the safety of the consumption of these products.

## Materials and Methods

### Ethical approval

Ethical approval was not needed for this study.

### Sample collection

A total of 44 samples from six most commonly consumed brands of commercial local liquid milk (raw, pasteurized, and UHT), labneh, and yogurt of different fat contents (whole/full fat, semi-skimmed/low fat, and skimmed/0% fat) that were commonly found on the shelves in main supermarkets in Keserwan region in Lebanon, one of the major districts in Lebanon, were purchased in May 2016. UHT milk samples were kept at room temperature, whereas all other samples were kept in the fridge at 4°C until analysis. All samples were analyzed before the expiration date.

### Dairy sample analysis using the enzyme-linked immunosorbent assay (ELISA) method

Both tetracycline and penicillin residue assays were carried out using ELISA test kit (R-Biopharm, Darmstadt, Germany), R3505 and R2921, respectively. Sample preparation and analysis were conducted according to the manufacturer’s instructions. Standard solutions of tetracycline and penicillin were provided in each test kit with concentration of 0, 0.15, 0.45, 1.35, and 4.05 ppb in aqueous solutions for tetracycline and from 0, 0.12, 0.25, 0.5, 1, 2, to 4 ppb for penicillin. The standard curves obtained experimentally were similar to those provided in the kits. This gives reliability to our experimental work, which was done by following the kit instructions, step by step.

For the quantitative estimation of tetracycline, samples were centrifuged at 3000 g for 10 min at 10°C for milk, 15 min at 4°C for labneh (following the cheese method provided by the ELISA kit), and 10 min at 10°C for yogurt. The upper cream layer was removed from the centrifuged samples, and they were diluted using the buffer provided in the kits. Milk, labneh, and yogurt samples were diluted to the ratio of 1:10, 1:5, and 1:10, respectively. For the quantitative estimation of penicillin, labneh and yogurt samples were initially centrifuged at 4000 g for 10 min at 4°C. After removing the upper cream layer, milk samples were diluted to the ratio 1:2 and labneh and yogurt samples were diluted to the ratio of 1:4 using the buffer provided in the kits.

For tetracycline analysis, 50 μL of each standard solution or prepared sample was added to each well followed by the addition of 50 μL of antibody. After incubation at room temperature (20-25°C) for 60 min, the wells were washed with the buffer twice, and then, 100 μL of the enzyme conjugate solution was added to each well. The plate was incubated at room temperature for 15 min. The last step of washing was required for all procedures and was repeated 2 times. Then, 100 μL of substrate was added to each well and incubated at room temperature for 15 min, in the dark. Finally, 100 μL of the stop reagent was added to each well, the absorbance was measured at 450 nm by a microplate reader (Thermo Labsystems Opsys MR, USA).

For penicillin analysis, 50 μL of the standard solution or prepared sample was added to each well. Then, 25 μL of conjugate was added followed by 25 μL of antibody. After incubation at room temperature for 60 min, the well was washed with the buffer twice, and then, 100 μL of the substrate chromogen solution was added and incubated at room temperature for 30 min, in the dark. Following the addition of 100 μL of the stop reagent to each well, the absorbance was measured at 450 nm by a microplate reader (Thermo Labsystems Opsys MR, USA).

The limit of detection (LOD) for tetracycline was 0.50 µg/kg for milk and 1.25 µg/kg for yogurt and labneh products, respectively. As for penicillin, the LOD for milk was 0.25 µg/kg and 1.25 µg/kg for yogurt and labneh products, respectively. Positive samples were defined by samples above the LOD for the test kits of tetracycline and penicillin residues.

### Assessment of the level of exposure to tetracycline and penicillin residues

The levels of exposure to tetracycline and penicillin residues were assessed using the below general equation. The average daily dairy intake as reported by Raad *et al*. [19] was multiplied by the mean residue concentration obtained in this study and divided by the average adult weight of 60 kg used as an estimator of the body weight (BW) [24]. The values were then summed up to reveal the level of exposure of the Lebanese population.

Estimated Daily Intake (µg/kg BW/day) = Σ [(Daily dairy intake (kg/person/day) × Mean residue concentration (µg/kg)] ÷ BW (kg).

The values obtained from this equation, which reveal the level of exposure of the Lebanese population to tetracycline and penicillin drug residues in all the dairy products were then compared to the acceptable daily intake (ADI) values set by CAC at a concentration of 0-30 μg/kg BW/day for each antibiotic in dairy products using the following equation:

% ADI = 100 × Intake (μg/day) ÷ [(ADI (μg/kg BW/day) × BW (kg)] [25].

### Statistical analysis

All samples were run in duplicate, and for the evaluation of test results, RIDA SOFTWIN software (Art. No. Z9999, R-Biopharm, Darmstadt, Germany) was used. The amount of antibiotic residues by processing status and fat content of milk, yogurt, and labneh were summarized as mean ± SD and median (interquartile range). The mean/median antibiotic residue content was based on positive samples. Negative samples were assigned a value of one half of the respective LOD when calculating the mean/median antibiotic residue concentration [26]. Percentages of detected positive samples in each of the dairy products were calculated. Statistical analyses were conducted using the Statistical Package for the Social Sciences Software version 22 (IBM, USA) and p<0.05 was considered statistically significant.

## Results and Discussion

The prevalence of samples detected positive for tetracycline and penicillin in milk, yogurt, and labneh with maximum, minimum, median, and average is reported in Table-1. The detection rate of tetracycline in milk (n=22) samples was 86.4% with a mean residues value of 1.16±0.70 μg/kg, respectively. The detection rate of tetracycline in labneh (n=12) and yogurt (n=10) samples was 50% for each with a mean value of 1.76±0.40 μg/kg and 0.63±0.12 μg/kg, respectively. As for penicillin residues, 90.9% of the milk (n=22) samples tested positive with a mean value of 0.52±0.25 μg/kg. The detection rate in labneh (n=12) and yogurt (n=10) samples was 0% for penicillin residues, where mean values were all below the LOD (<1.25 μg/kg) for these dairy products.

**Table-1:** Number of samples detected positive for tetracycline and penicillin in milk, yogurt, and labneh with their minimum, maximum, median, and mean values.

Dairy product	Tetracycline						Penicillin					
	
	Positive^a^/n	% positive	Minimum/maximum of the positive samples (μg/kg)	Median (μg/kg)	Interquartile range	Mean±SD (μg/kg)	Positive^a^/n	% positive	Minimum/maximum of the positive sample (μg/kg)	Median (μg/kg)	Interquartile range	Mean±SD (μg/kg)
Milk	19/22	86.4	0.58/3.33	0.80	1.08	1.16±0.70	20/22	90.9	0.31/0.92	0.47	0.25	0.52±0.25
Yogurt	5/10	50.0	0.50/0.76	0.63	0.11	0.63±0.12	0/10	0	0	-*	-*	0
Labneh	6/12	50.0	1.39/2.32	1.01	1.04	1.76±0.40	0/12	0	0	-*	-*	0

a Positive samples: Samples in which the antibiotic residue levels exceeded the LOD. LOD tetracycline: 0.50 mg/kg for milk and 1.25 mg/kg for yogurt and labneh products. LOD for penicillin: 0.25 mg/kg for milk and 1.25 mg/kg for yogurt and labneh products.

* Amount of penicillin residue is constant. It has been omitted. LOD=Limit of detection

Table-2 shows the percentages and levels of positive samples for tetracycline and penicillin in different milk samples obtained by various thermal processing techniques (raw, pasteurized, and UHT) and with variable fat content (full fat, half skimmed/low fat, and skimmed/0% fat). The results show that 100% of the raw (n=4) and pasteurized (n=9) milk samples contained tetracycline residues, while 66.7% of the UHT (n=6/9) were positive. Among all these milk samples, 90% of the full fat milk (n=9) were positive with a mean value of 1.29±0.88 μg/kg followed by 83.3% of each of half skimmed/low fat (n=5) and skimmed/0% fat samples (n=5) with a mean value of 1.17±0.55 μg/kg and 0.93±0.49 μg/kg, respectively, whereas 75.0%, 88.9%, and 100% tested positive for penicillin residues in raw (n=4), pasteurized (n=9), and UHT (n=9) milk samples. Among the positive milk samples of different fat content, full fat milk (n=9) had the highest detection rate of 40.9% and a mean value of 0.46±0.23 μg/kg followed by 27.3% of half skimmed/low fat milk (n=5) with a mean value of 0.74±0.15 μg/kg and 22.7% of skimmed/0% fat (n=5) with a mean value of 0.46±0.31 μg/kg. Tetracycline and penicillin residues were found to be below the MRL set by the EU Union and the CAC of 100 μg/kg and 4 μg/kg, respectively, in all dairy products.

**Table-2:** Number of samples detected positive for tetracycline and penicillin in different milk samples.

Dairy product	Tetracycline						Penicillin					
	
	Positive^a^/n	% positive	Minimum/maximum of the positive sample (μg/kg)	Median (μg/kg)	Interquartile range	Mean±SD (μg/kg)	Positive^a^/n	% positive	Minimum/maximum of the positive sample (μg/kg)	Median (μg/kg)	Interquartile range	Mean±SD (μg/kg)
Raw milk	4/4	100.0	0.66/1.71	0.80	0.83	0.99±0.49	3/4	75.0	0.31/0.68	0.38	0.45	0.48±0.19
Pasteurized milk	9/9	100.0	0.58/1.75	1.04	0.85	1.06±0.45	8/9	88.9	0.38/0.92	0.65	0.34	0.64±0.18
UHT milk	6/9	66.7	0.58/3.33	0.62	1.46	1.43±1.07	9/9	100.0	0.35/0.60	0.46	0.16	0.47±0.09
Full fat milk	9/10	90.0	0.58/3.33	0.99	1.65	1.29±0.88	9/22	40.9	0.31/0.80	0.45	0.27	0.46±0.23
Half skimmed milk	5/6	83.3	0.58/1.76	0.93	1.18	1.17±0.55	6/22	27.3	0.41/0.92	0.60	0.28	0.74±0.15
Skimmed milk (0% fat)	5/6	83.3	0.58/1.74	0.64	0.72	0.93±0.49	5/22	22.7	0.41/0.73	0.50	0.30	0.46±0.31

a Positive samples: Samples in which the antibiotic residue levels exceeded the LOD. LOD tetracycline: 0.50 mg/kg for milk and 1.25 mg/kg for yogurt and labneh products. LOD for penicillin: 0.25 mg/kg for milk and 1.25 mg/kg for yogurt and labneh products. LOD=Limit of detection

In Kuwait, 45 of 121 (37.0%) raw milk samples and 22 of 306 (7.0%) imported pasteurized milk samples had tetracycline residues above the MRL [14]. Of the 22 imported pasteurized milk samples with tetracycline residues above MRL, 14 (63.6%) were full fat, 5 (22.7%) were low fat, and 3 (13.6%) were skimmed milk. As for the local pasteurized milk samples, results showed that 16 of the 208 samples (8%) had tetracycline residues above the MRL with 7 (43.7%) being of full fat, 4 (25.0%) of low fat, and 5 (31.2%) of zero fat content. Although results reported in Al Mazeedi *et al*. [14] were above MRL for tetracycline residues, full fat pasteurized milk contained a higher percentage of residues, followed by low fat and skimmed milk which is similar to our findings reported in Table-2.

The prevalence of all contaminated samples in our study was found to be high, and the rate was higher for both antibiotics in milk samples compared to labneh and yogurt samples. This rate is even higher than other neighboring countries such as Palestine which had a contamination rate of 22.2% (n=4 of 18) for β-lactams and 18.7% (n=3 of 16) for tetracycline in raw milk samples [1]. Moreover, when compared with the Chinese market, 0% detection percentage for tetracycline residues in UHT milk samples (n=180) was reported [26]. This discrepancy in the findings could be attributed to the prolonged and excessive use of antibiotics among farmers reported earlier [23]. The decreased contamination rate of these residues in labneh and yogurt samples could indicate that food production industries are aware of the manufacturing problem that could lower these products quality since the presence of antibiotic residues may interfere with dairy cultures [27].

The low levels of antibiotic residues in pasteurized and UHT milk samples reported in our study could be due to the low heat stability of tetracycline and penicillin [28]. For instance, the thermostability of tetracycline resulted in 98% destruction after analysis of 94 UHT milk samples in China [7]. Moreover, penicillin G is found to be the most heat sensitive compared to OTC and tetracycline [28] which can be explained by the findings of this study where the mean value of penicillin was lower in milk samples compared to the mean value of tetracycline in the same samples with 0.52±0.25 μg/kg versus 1.16±0.70 μg/kg, respectively. Moreover, no penicillin residue was detected in labneh and yogurt samples.

Furthermore, our study confirms the fact that both antibiotics are not fully eliminated after processing and that traces of residues would still be detected following heat treatment. Similarly, tetracycline residues were present in all yogurt samples (n=8) after processing of cow’s milk in Nigeria with levels < MRL [29]. It is important to point out, that in our study, the comparison of residues by processing technique and fat content should be interpreted with caution since skimmed/processed dairy samples were not derived from the same batch of full fat/raw samples.

The results of this study are in line with the previous studies done in China, Turkey, and Croatia [26,30]. For instance, tetracycline residues using ELISA in commercial UHT milk samples in China (n=180), and tetracycline levels were found to be less than LOD of 1.5 µg/kg for all samples [26]. Furthermore, using ELISA, tetracycline levels in UHT milk samples (n=60) in Turkey were found in 66.8% of the samples with a mean value of 1-3.3 µg/kg [30].

On the contrary, other studies [1,15,31] detected antibiotic residues > MRL for both antibiotics. For instance, a study conducted in Palestine to assess tetracycline and β-lactams residues showed that 3 of 16 (18.7%) and 4 of 18 (22.2%) raw milk samples contained tetracycline and β-lactams residues above the MRL, respectively [1]. Another study conducted in Iran on 208 local pasteurized milk samples found that 17 samples (8%) had tetracycline residues above the MRL ranging in an amount between 100 and 230 µg/kg [15]. Furthermore, a study done in India on 133 raw cattle milk samples showed that 13.5% of the samples contained tetracycline residues with concentrations ranging between 16 and 134.5 µg/kg, of which 2.3% (n=3) exceeded the MRL for tetracycline [31].

It is very likely that the level of residues reported in our study was below the MRL due to several reasons. For instance, we limited our study to major dairy farms/industries which are applying extensive quality control procedures, especially on the initiation of the Lebanese food safety campaign by the Ministry of Health in November 2014 which is still ongoing till present. Moreover, five of six brands have a basic quality assurance program that complies with the Lebanese and international standards, one of which is ISO 22000:2005 certified for Food Safety Management System. Hence, these dairy products should be routinely tested for antibiotic residues and other contaminants in these industries before dispatch. On the other hand, it would be interesting to assess antibiotic residues in small industries in Lebanon. It is very likely that higher antibiotic residue levels would be observed in such industries due to lower quality control measures in such settings, as well as lack of farmer’s knowledge and poor management controls on the administration of antibiotics, as it was observed in small dairy farms in Peru [16].

Furthermore, it is important to highlight that samples in the current study were collected in May, and in Lebanon, this month falls in the spring season during which a lower incidence of mastitis and other diarrheal diseases is likely to occur. The highest incidence of mastitis was seen during the winter season (December-March), requiring longer treatment duration and increasing the prevalence of detecting antibiotic residues in food products [16,32].

Moreover, another study showed that the number of samples of the raw and pasteurized milk that tested positive for antibiotic residues was found to be the highest in January of the winter season [18]. It is suggested that both in winter and summer seasons, treatment efficacy had adverse effects due to cold/heat stress on treatment outcome [32]. This theory is supported by a study conducted during the period of January-July in Iran, where 187 UHT and pasteurized milk samples were assessed, and it was found that in the warm season of the year, along with increase in weather temperature, the prevalence of contaminated samples was raised which is suggested to be due to higher incidences of diarrheal diseases, which result in the administration of antibiotics to cattle [15]. Moreover, the amount of the residues of both tetracycline and penicillin was noticeably low in May. Although there was no significant difference (p<0.05) between the contamination rate between cold (January-March) and warm season (April-July) in the assessed samples, the distribution of contamination rate showed that highest contamination occurred in February (45%) followed by July (41%) and June (40%). Accordingly, it would be of high interest to reassess antibiotic residues in dairy products in Lebanon during other seasons.

The EDI of tetracycline and penicillin residues obtained from consumption of each dairy product, as well as the percent ADI is presented in Table-3 [15,19]. The EDI of tetracycline residues calculated was 1.28 ng/kg BW/day, 0.43 ng/kg BW/day, and 0.38 ng/kg BW/day, respectively, for each of the milk, yogurt, and labneh. It is important to note that pasteurized and UHT milk samples were taken into consideration when assessing the antibiotic residue intake of Lebanese population because raw milk is not consumed as such. The estimated dietary intake (EDI) is, therefore, 2.09 ng/kg BW/day resulting in 0.007% of the ADI. Expressing the dietary intake of tetracycline as a percentage of ADI resulted in an ADI of 0.004% for milk, and 0.001% for each of the yogurt and labneh (Table-3).

**Table-3:** Risk characterization of daily exposure to tetracycline and penicillin residues through intake of each of the dairy products individually as well as through intake of a combination of all dairy products.

Reference	Antibiotic	Milk			Yogurt			Labneh			Total dairy products	
			
		Intake (g milk/day)	EDI^a^	% ADI^b^	Intake (g yogurt/day)	EDI^a^	% ADI^b^	Intake (g labneh/day)	EDI^a^	% ADI^b^	Total EDI^a^	% ADI^b^
Raad *et al*. [19]	Tetracycline	99.7	1.28	0.004	68.3	0.43	0.001	27.8	0.38	0.001	2.09	0.007
	Penicillin	99.7	0.83	0.003	68.3	0.71	0.002	27.8	0.29	0.001	1.83	0.006

a EDI=Estimated daily intake (ng/kg BW/day). EDI (mg/kg BW/day)=S[(Daily dairy intake (kg/person/day)×Mean residue concentration (mg/kg)] ¸ BW (kg).

b % ADI=100×Intake (mg/day)/[(ADI (mg/kg BW/day)×BW (kg)] [15]. ADI=Acceptable daily intake (Joint Food and Agriculture Organization/World Health Organization Expert Committee for Food Additives) for each of the antibiotics: ADI_Tetracycline/Penicillin_=30 mg/kg BW.

In contrast to the findings of our study pertaining to the percentage ADI, the dietary exposure to tetracycline residues through milk consumption for Iranian adult (>18 years) consumers reached up to about 6% of the ADI at short-term exposure, a percentage higher than the findings of our study due to the fact that the average concentration of all tetracyclines in milk reported in this study reached approximately 2.5 times greater than the MRL [25].

As for penicillin residues, for milk, yogurt, and labneh products, the EDI for penicillin residues through consumption of these dairy products was 0.83 ng/kg BW/day, 0.71 ng/kg BW/day, and 0.29 ng/kg BW/day, respectively, which constituted 0.003%, 0.002%, and 0.001% of the ADI (Table-3). The EDI is, therefore, 1.83 ng/kg BW/day for all dairy products which resulted in 0.006% of the ADI. In Croatia, the EDI for β-lactams ranged between 1% and 5% of the ADI depending on the estimated intake [33]. In our study, the percentage ADI could be due to the fact that all yogurt and labneh samples which constitute half of the tested samples had no detected contamination rate for penicillin and values were below the LOD.

Although all EDI values were below the ADI set for each antibiotic residue and do not exceed relevant toxicological reference values, low levels of antibiotic residues could still pose an adverse health risk to humans. Approximately 4-11% of the human populations are believed to be allergic to penicillin manifested by minor reactions such as skin rash to severe anaphylaxis [34]. It is believed that 10 IU (0.6 μg) or 6 ng/ml of penicillin residue in milk can cause this reaction [34]. In our study, mean penicillin residue level was found to range between 0.52±0.25 μg/kg and 0.92 μg/kg as a maximum level found in pasteurized half skimmed milk samples (Tables-2 and 3). Furthermore, concentrations as low as 0.006 µg (0.01 IU) penicillin per gram of milk considerably inhibit starter cultures and delay acid production. Penicillin residues must not exceed 0.003 µg (0.005 IU) per gram of milk to get an acceptable quality of dairy product [35]. Moreover, the presence of penicillin in consumed meat was detected, and evidence of an adverse drug reaction was provided by testing patient sensitivity to penicillin [34]. On the contrary, there is no reported evidence to date of human consumption of animal products containing tetracyclines having resulted in adverse reactions related to what is associated with tetracycline toxicity [34]. However, long-term effects arising from the intake of other products of animal origin that has been contaminated with antibiotic residues have been suggested [34]. It is important to note that the effect of combining very low concentrations of one single antibiotic or a combination of two or more antibiotics, especially with heavy metals can still result in antibiotic resistance [36]. Thus, it could allow for the selection of bacteria with multiresistance and thereby contribute to the emergence, maintenance, and transmission of antibiotic-resistant disease-causing bacteria [36].

## Conclusion

The overall results showed that the prevalence of tetracycline and penicillin residues in milk, yogurt, and labneh was high in terms of contamination among the collected samples although their levels were below MRL. The estimated daily intake of the Lebanese consumers was below ADI; however, the low levels of antibiotic residues could still pose adverse health risks. Our findings highlight the need for antibiotic monitoring, particularly at different seasons, among different food products and among small dairy industries that do not necessarily apply stringent quality control measures. Findings of this study highlight that continuous monitoring is needed to prevent adverse health reactions that can still occur among vulnerable Lebanese consumers, especially those who are hypersensitive, making food safety a challenging issue to the manufacturers and food authorities.

## Authors’ Contributions

SK: Collected and analyzed data and drafted the manuscript. HFH: Conceptualized and designed the study/Co-wrote the manuscript. JMBM: Designed the analysis of the study and carried out the statistical analysis. CB: Analyzed data and reviewed the final draft. JEF: Drafted the manuscript and reviewed the final draft. All authors read and approved the final manuscript.
